# Case report: Alectinib-associated intestinal ulceration and colitis in a patient with non-small cell lung cancer and effective treatment with Mesalazine

**DOI:** 10.3389/pore.2025.1612040

**Published:** 2025-03-06

**Authors:** Zijian Qiu, Fei Ke, Xiaoping Zhu

**Affiliations:** ^1^ Department of Radiation Oncology, The Quzhou Affiliated Hospital of Wenzhou Medical University, Quzhou People’s Hospital, Quzhou, Zhejiang, China; ^2^ Department of Pathology, The Quzhou Affiliated Hospital of Wenzhou Medical University, Quzhou People’s Hospital, Quzhou, Zhejiang, China

**Keywords:** Alectinib, intestinal ulcer, colitis, anaplastic lymphoma kinase inhibitor, non-small cell lung cancer

## Abstract

**Background:**

Alectinib is effective in extending the survival of patients with anaplastic lymphoma kinase (ALK)-positive non-small cell lung cancer (NSCLC) and generally has manageable side effects. However, intestinal ulcers and colitis are rare but serious adverse reactions linked to Alectinib, meriting further investigation into their causes.

**Case presentation:**

We report the case of a 62-year-old woman with NSCLC and brain metastases, who tested positive for ALK. She had been treated with Alectinib for nearly 4 years. The patient experienced diarrhea for 4 days, and a subsequent colonoscopy revealed pancolitis along with multiple ulcers in the terminal ileum and ileocecal valve. Given the severity of these symptoms, classified as a grade 3 adverse event by the Common Terminology Criteria for Adverse Events (CTCAE), Alectinib was discontinued. Treatment with oral enteric-coated Mesalazine tablets led to a resolution of the diarrhea and a significant improvement in the pancolitis and ulcers upon follow-up. The patient’s anticancer therapy was subsequently switched to Ceritinib capsules. At follow-up, she demonstrated a stable tumor condition with no recurrence of intestinal ulcers or colitis.

**Conclusion:**

To our knowledge, this is the first reported case of intestinal ulceration and colitis induced by Alectinib. Although such adverse events are exceedingly rare, they require vigilant monitoring in clinical practice. Decisions on continuing with Alectinib should consider the severity of side effects, classified by CTCAE grade. For managing these specific adverse events, oral Mesalazine enteric-coated tablets appear to be an effective treatment option.

## Introduction

ALK gene mutations are significant drivers in the development of NSCLC, though they occur in only about 2%–7% of cases, often as echinoderm microtubule-associated protein-like 4 (EML4)-ALK fusions [1, 2]. Various ALK inhibitors against this target (including Crizotinib, Alectinib, Ceritinib, and Lorlatinib) have been approved for use [3] and developed into a fourth-generation drug [4]. Among these, Alectinib serves as a first-line treatment for advanced ALK-positive NSCLC. The ALEX study reported a median progression-free survival of 34.8 months and a 5-year overall survival rate of up to 62.5% [5], showcasing mild adverse effects and a robust safety profile [3, 6]. Common side effects of Alectinib include constipation, muscle pain, edema, elevated bilirubin, anemia, and rash [7]. However, there have been rare reports of severe adverse events such as osteonecrosis of the femoral head [8], chylothorax [9], acute renal failure [10], hemolytic anemia [11], drug-associated pneumonia [12], and gastrointestinal perforation [13, 14]. This case marks the first report of intestinal ulceration and colitis during treatment with Alectinib. It also outlines the application of an enteric-coated Mesalazine tablet regimen and assesses its effectiveness.

## Case description

**FIGURE 1:**
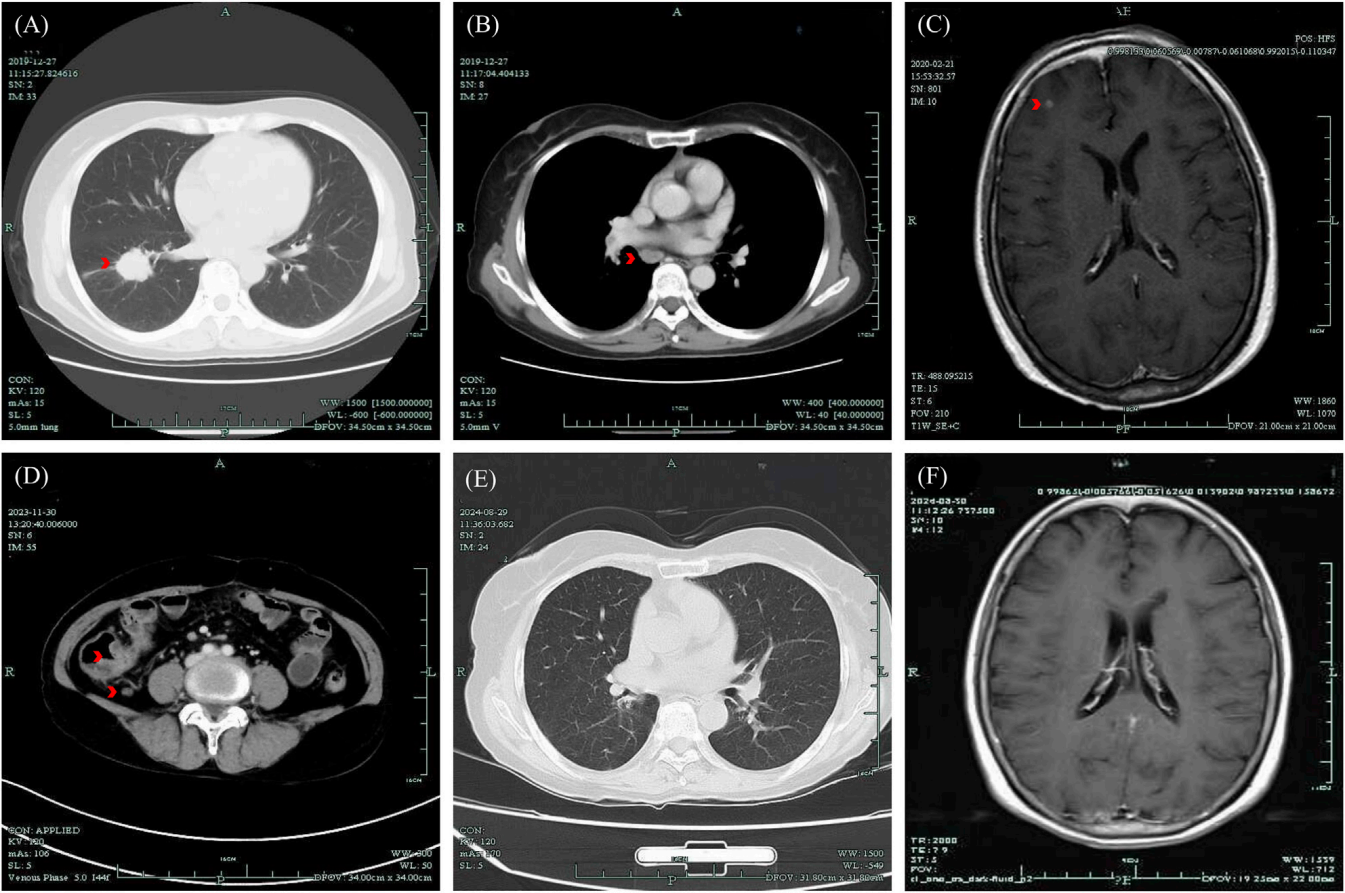
Medical images presented based on the clinical course of the patient. **(A, B)** CT image of lung lesion and enlarged mediastinal lymph nodes, December 2019; **(C)** MR image of brain metastases found post-operatively, February 2020; **(D)** CT image of thickening of the ileocecal junction, December 2023; **(E)** CT image of the chest with no tumor recurrence, 29 August 2024; **(F)** MR image of persistent complete remission of the intracranial lesion, August 29, 2024. CT, Computed Tomography; MR, Magnetic Resonance.

The Chinese female patient, with no history of chronic disease or long-term drug use, was found to have a right lung lesion and enlarged hilar and mediastinal lymph nodes during a physical examination at 59 years old (December 2019), Computed Tomography (CT) images are shown in Figures 1A, B. She underwent pneumonectomy and mediastinal lymph node dissection. The postoperative pathology revealed poorly differentiated adenocarcinoma with slight squamous differentiation (tumor size: 2.5 cm × 2.5 cm × 2.0 cm), bronchus invasion (bronchial margins negative), nerve invasion, and positive lymph nodes at Station 7 (3/3), Station 9 (1/1), and Station 10 (1/1). Genetic testing of the tumor specimen identified an EML-4 (Exon 13)-ALK (Exon 20) fusion mutation. The initial staging was pT1cN2Mx, and the patient was scheduled for postoperative adjuvant therapy in January, when a follow-up review revealed multiple small nodules in the right frontal region (Figure 1C), indicating brain metastases. Consequently, her staging was revised to stage IVB. She received Alectinib treatment for nearly 4 years, during which intracranial lesions resolved and no signs of tumor recurrence in the chest were observed. The treatment efficacy was evaluated as complete remission.

**FIGURE 2:**
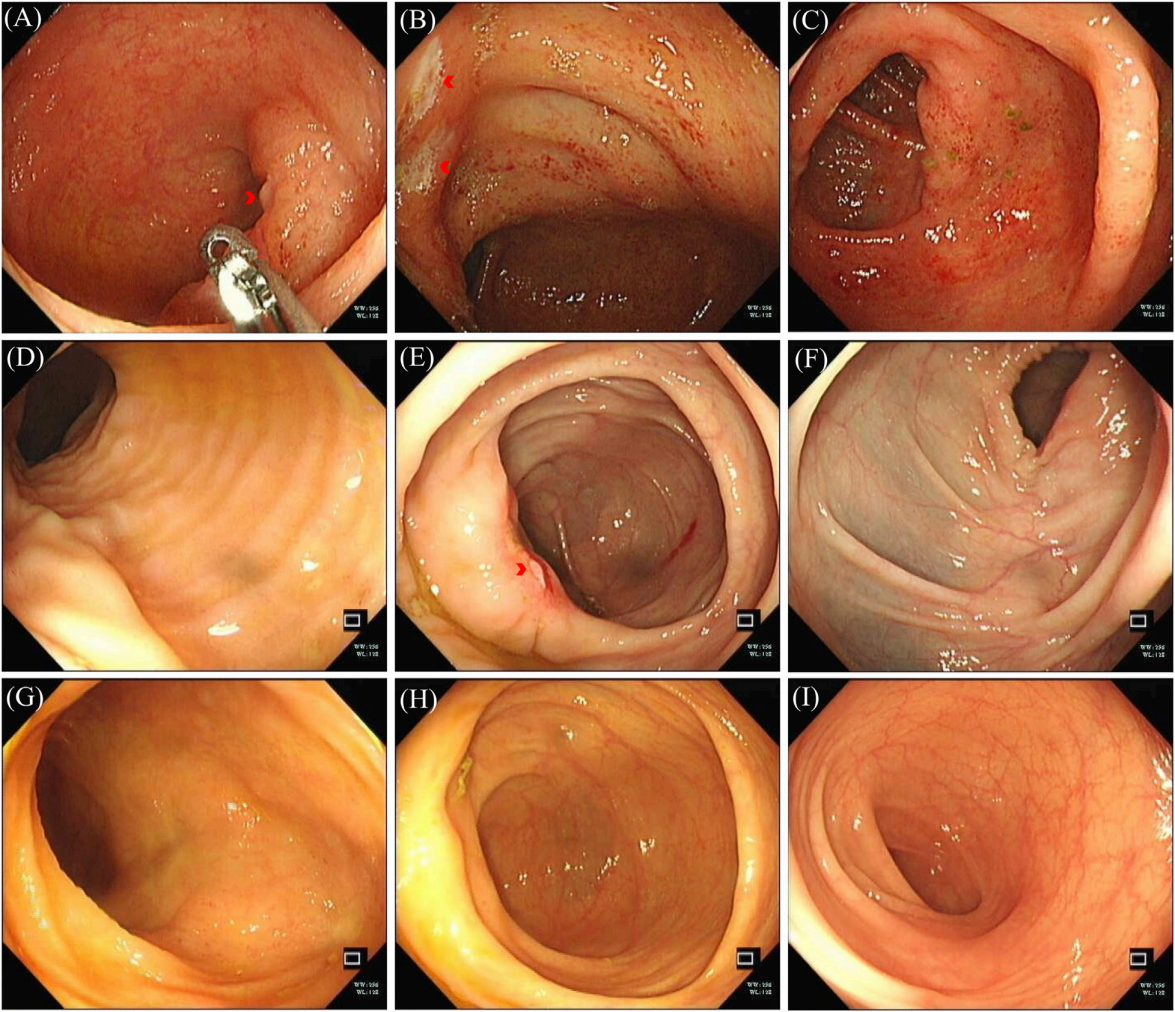
Colonoscopy image of colonic lesions. **(A–C)** Colonoscopy images, December 2023: **(A)** terminal ileum ulceration; **(B)** ileocecal valve ulceration; **(C)** swollen and congested colon. **(D–F)** Colonoscopy images, January 2024: **(D)** no ulceration at terminal ileum; **(E)** ileocecal valve ulceration; **(F)** colon congestion and swelling subsided. **(G–I)** Colonoscopy, September 2024; no pathological changes seen in colon and terminal ileum: **(G)** terminal ileum; **(H)** ileocecal junction; **(I)** colon without congestion.

**FIGURE 3:**
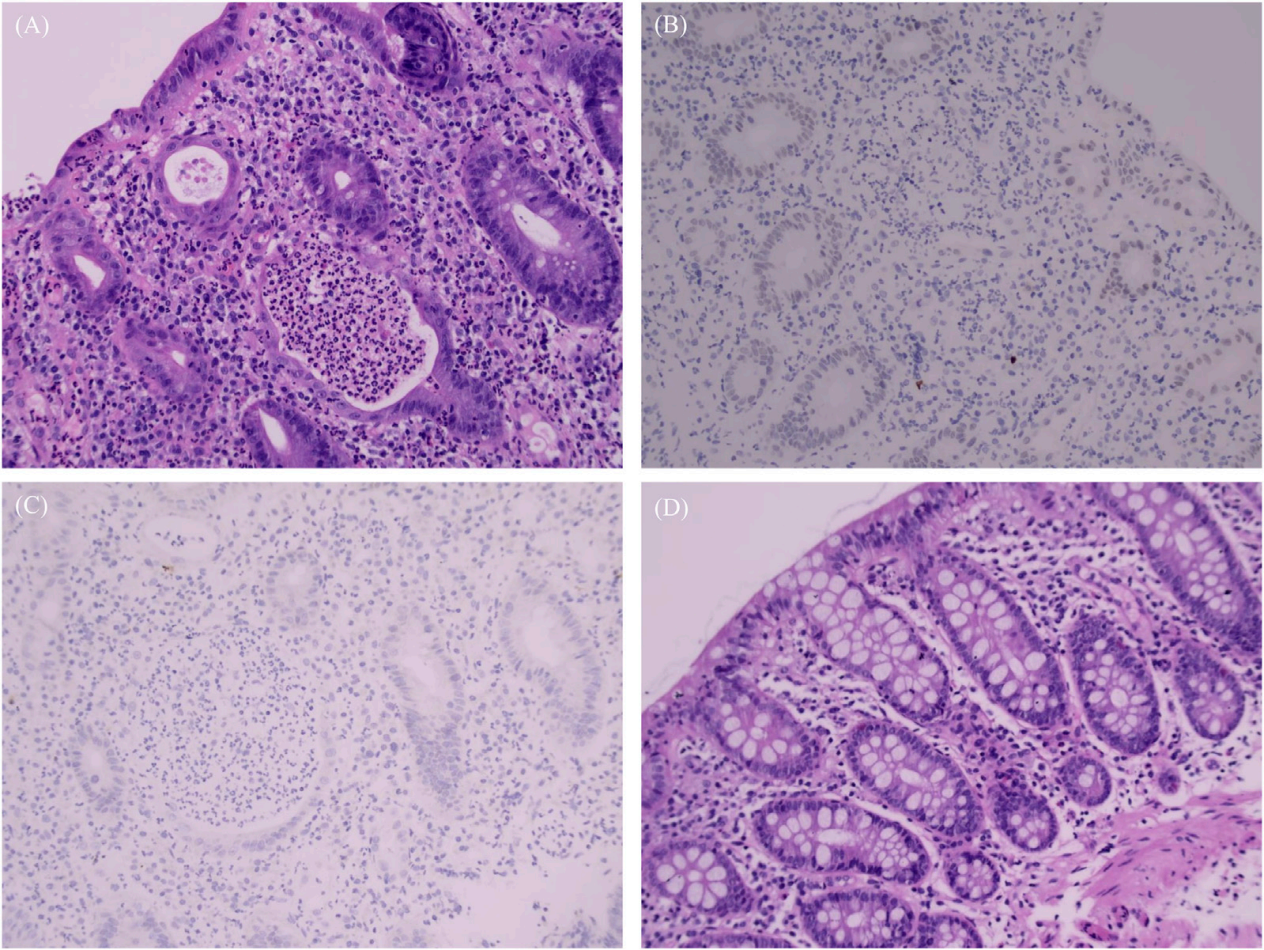
Biopsy images. **(A–C)** Pathology and immunohistochemistry results, December 2023: **(A)** Severe active ulceration in the colon mucosa featuring twisted, atrophied, and flattened crypts; cryptitis, crypt abscesses; presence of apoptotic bodies in the crypt epithelium and increased infiltration of lymphocytes; **(B)** Immunohistochemistry: CMV negative; **(C)** EBER negative. **(D)** Pathology, September 2024: colonic mucosa with preserved crypt structures and no active inflammation.

The patient sought medical attention on 30 November 2023, with symptoms of “diarrhea for 4 days” characterized by yellow, loose, watery stools occurring about ten times daily and slight tenderness in the right lower abdomen. There were no signs of rebound tenderness, dehydration, fever, abdominal pain, nausea, vomiting, blood in the stool, or black stool. The patient reported no recent unclean diet or medications other than Alectinib. Upon admission, immediate examinations were conducted. Routine stool tests were negative for red blood cells and leukocytes, and the fecal occult blood test was also negative. Stool cultures showed no growth of *Shigella* or *Salmonella*. Hemoglobin levels were at 92 g/L, and leukocyte count was 4.4 × 10^9^/L. An enhanced CT scan of the abdomen indicated thickening of the bowel wall and mild enlargement of multiple surrounding lymph nodes (Figure 1D). Colonoscopy revealed multiple ulcers in the terminal ileum, ileocecal valve, and signs of pancolitis (Figures 2A–C). Gastroscopy identified chronic non-atrophic gastritis. A biopsy indicated increased infiltration of lymphocytes in the epithelial layer of colonic mucosa and the presence of apoptotic bodies in the crypt epithelium, with negative tests for Cytomegalovirus (CMV) and Epstein-Barr virus-encoded RNA (EBER), the pathological images are shown in Figures 3A–C. Based on these findings, infectious diarrhea and intestinal tumors were ruled out, confirming a diagnosis of intestinal ulceration and colitis linked to ALK inhibitors, classified as a CTCAE grade 3 adverse event. Alectinib was discontinued, and the patient was prescribed oral enteric-coated Mesalazine tablets, taken three times daily at 1.2 g each. Additionally, oral capsules containing Live Combined Bifidobacterium, *Lactobacillus*, and *Enterococcus* were administered. The patient’s diarrhea symptoms receded and disappeared. Seven weeks later, a follow-up colonoscopy showed the colitis had subsided and the terminal ileum ulcer had healed (Figures 2D–F). After 12 weeks, the patient did not show tumor progression and transitioned to oral Ceritinib capsules to continue antitumor therapy. Nine months later, the ileocecal valve ulcer was reviewed and had completely healed (Figures 2G–I, 3D). To date, the patient has maintained a stable tumor condition in follow-ups without any recurrence of intestinal ulcers or colitis (Figures 1E, F).

## Discussion

ALK inhibitors, small molecule drugs targeting the ALK gene, are the first-line treatment for patients with ALK-positive advanced NSCLC. Alectinib, in particular, improves post-surgical disease-free survival for patients with stage IB, II, or IIIA NSCLC and is recommended for postoperative adjuvant therapy [15]. Although ALK inhibitors are generally well tolerated and provide clinical benefits, their adverse effects remain a concern. Meta-analyses have shown varying toxicity profiles among different ALK inhibitors, with gastrointestinal reactions being the most common. For instance, diarrhea is the most reported adverse effect of Crizotinib, Brigatinib, and Ceritinib, while constipation is most frequently associated with Alectinib [16]. These gastrointestinal adverse reactions are typically mild to moderate (CTCAE grades 1-2) and manageable. However, Alectinib has the lowest incidence of severe (grade 3-4) AEs, though cases of gastrointestinal perforation have been reported [13, 14]. Such incidents underscore the importance of not overlooking rare and severe intestinal adverse reactions.

The mechanism by which ALK inhibitors induce intestinal ulceration and colitis remains unclear. ALK is primarily expressed in the nervous system, small intestine, and testes [17]. It is also present in the gut of *Drosophila* embryos [18]. In addition to NSCLC, the ALK fusion gene [19] is also present in colorectal cancer. Another targeted drug, Dacomitinib, inhibits various kinases in the ErbB family. It was observed that ErbB1 is highly expressed in the ileum of rats, suggesting that Dacomitinib may induce severe damage to the ileum and alter gastrointestinal permeability, leading to diarrhea [20]. Therefore, organ damage may be linked to the high expression of the targeted receptor in the affected organ.

In our case, apoptotic bodies and damaged crypts were observed in the affected intestinal tissues. Apoptotic bodies are small vesicles that form when cells undergoing apoptosis are engulfed by phagocytes, and an increase in these bodies suggests either a heightened rate of apoptosis or a reduced clearance of apoptotic vesicles. This can trigger the secretion of pro-inflammatory cytokines and disrupt the immune system [21]. Additionally, structural changes in the crypts, such as distortion, atrophy, and flattening, are pathological features of intestinal inflammation. We also performed immunohistochemistry tests for cytomegalovirus and EBV, which returned negative, excluding associated viral infections. Therefore, the intestinal pathological changes were attributed to the effects of immunomodulators, as ALK inhibitors can modulate immune functions [22] alongside their antitumor properties. These findings support the diagnosis of ALK inhibitor-associated intestinal ulcers and colitis in this patient, suggesting that the mechanism behind this intestinal pathology may be autoimmune-related.

Studies have shown that mental disorders are also important causes of ulcerative colitis, especially in elderly women and cancer patients [23]. Mental health problems are an easily overlooked factor that needs to be taken seriously and differentiated for diagnosis. In our case, this patient did not show clinical signs of anxiety or depression, but mental health consultation and evaluation were missing, which is a shame. However, we still recommend that evaluation and identification of psychiatric disorders is necessary when patients have gastrointestinal adverse reactions.

The expert consensus for managing adverse reactions to ALK inhibitors recommends using the CTCAE to assess the severity of an adverse event and guide treatment decisions [24]. Currently, there is no established pharmacological treatment for ALK inhibitor-associated intestinal ulcers and colitis due to the rarity of this condition. Drawing on treatments for similar pathologies, we used enteric-coated Mesalazine tablets, an aminosalicylic acid drug, following protocols for ulcerative colitis. The use of glucocorticoids remains controversial; they may be suitable for patients with severe illness or those unresponsive to aminosalicylic acid drugs. For adverse events of CTCAE grade 3 or higher, discontinuation of the original antitumor drug is necessary. Thus, we discontinued Alectinib and switched the patient to another ALK inhibitor, Ceritinib, to continue her antitumor therapy. Although this patient has been safely treated with Ceritinib for 8 months, the potential recurrence of intestinal disease necessitates regular follow-up. Gastrointestinal adverse effects continue to be the predominant issues with ALK inhibitors, and it is hoped that emerging targeted therapy drugs will reduce the incidence of such side effects [16].

## Conclusion

Alectinib-induced intestinal ulcers and colitis are extremely rare adverse reactions. Patients should be vigilant for such events during treatment with ALK inhibitors. When gastrointestinal symptoms arise, prompt colonoscopy and pathologic diagnosis are crucial. The CTCAE should be employed to assess the severity of the condition and help formulate a treatment plan. Discontinuing the initial ALK inhibitor and starting treatment with enteric-coated Mesalazine tablets has proven to be an effective approach. It remains uncertain whether switching to another ALK inhibitor will lead to a recurrence of intestinal ulcers and colitis.

## Data Availability

The original contributions presented in the study are included in the article/supplementary material, further inquiries can be directed to the corresponding author.
